# Supplementary material for: Mistranslating tRNA variants have anticodon- and sex-specific impacts on Drosophila melanogaster
Source: G3 (Bethesda). 2024 Sep 23;14(12):jkae230. doi: 10.1093/g3journal/jkae230 (PMC11631534; doi:10.1093/g3journal/jkae230)
Supplement: jkae230_Supplementary_Data [file jkae230_supplementary_data.zip › File_S2_G3-2024-405333.docx]

## Supplemental Information

Extended Methods

*Scoring for eye degeneration*

Equal numbers of male and female adults containing a mistranslating tRNA^Ser^ variant or its corresponding control were collected within 8 hours of eclosion and aged 30 days. Flies were transferred to new food every three days. Upon reaching 30 days of age, fly heads were removed and immobilized in Blu-Tack (Bostik, Ltd.) with their left eye pointing upwards. The left eye was imaged at 10x magnification on a Zeiss Axio Imager Z1 Fluorescent microscope using ZEN Blue Pro software (v3.1, Zeiss Inc.) and ~25 images 4.08 µm apart were combined using focus stacking to produce the final image. A circle 15 ommatidia in diameter was drawn on the centre of the eye and the number of pixels within recorded. The number of pixels within that region corresponding to degenerated regions of the eye were recorded and used to calculate the percentage of the eye that showed signs of neurodegeneration. Prevalence of neurodegeneration in the different lines was compared using Wilcoxon rank-sum tests corrected using Holm-Bonferroni’s method.

*GO analysis of codon and amino acid abundance in the Drosophila melanogaster proteome*

For each gene, the number of a specific codon or amino acid was calculated using a custom perl script. Genes were ranked based on the proportion of a specific codon or amino acid within the gene. GO analysis was performed on the ranked list using Gorilla (Eden et al. 2009). GO terms from the bioprocess, component and function term were filtered for statistically significant terms with FDR Q-value less than 0.01 and enrichment score greater than 3. Semantically redundant terms were removed.

Table S1: Primers used.

| Primer name | Sequence |
| --- | --- |
| tSer_US | GGTATGAAGCATAGATTTCAGC |
| tSer_DS | CCCGCACGGGAAATTCCTAGG |
| tSerAAC_F | AGGAGATGGACTAACAATCCATTGGGTTCTAC |
| tSerAAC_R | CCCAATGGATTGTTAGTCCATGTCCTTAACCA |
| tSerAGU_F | AGGAAATGGACTAACAATCCATTGGGTTCTAC |
| tSerAGU_R | CCCAATGGATTGTTAGTCCATTTCCTTAACCA |
| pattB_tRNA_Ver_F | GGATTTCACTGGAACTAGGC |
| pattB_tRNA_Ver_R | CCTACATCGTCGACACTAGT |
| FRT-tRNA-Ver_F | GGTGGGCATAATAGTGTTGTTTAT |
| FRT-tRNA-Ver_R | CTAGAGGTACCCTCGAGCCG |

Table S2: Calculating expected mistranslation rate at each codon. Cognate codons for the tRNA^Ser^ variants used in this study are bolded. Number of competing tRNAs were obtained from GtRNAdb (Chan and Lowe 2016), and include the single copy of variant tRNA^Ser^ and tRNAs that can decode the listed codon through wobble or superwobble.

| Codon | Codon usage (A)^a^ | # of competing tRNAs (B) | Mistranslation rate (A/B, %) |
| --- | --- | --- | --- |
| **Valine** |  |  |  |
| **GUU** | 0.18 | 17 | 1.06 |
| GUC | 0.24 | 17 | 1.41 |
| GUA | 0.11 | 17 | 0.65 |
| **Threonine** |  |  |  |
| **ACU** | 0.17 | 29 | 0.59 |
| ACC | 0.38 | 29 | 1.31 |
| ACA | 0.19 | 29 | 0.66 |

^a^Codon usage information was obtained from [https://www.genscript.com/tools/codon-frequency-table](about:blank).


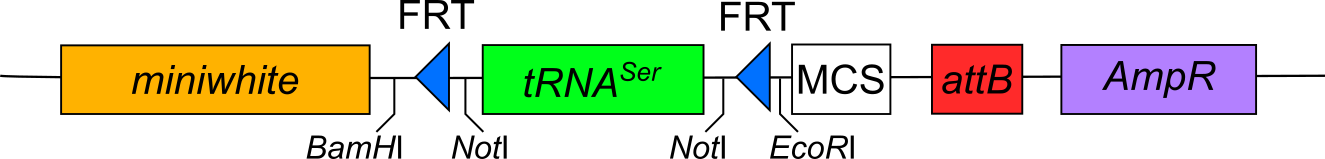


Figure S1. *Schematic of pattB-ΔNotI/pUCIDT.* Order of key elements of the pattB-Δ*Not*I/pUCIDT plasmid used to integrate tRNA^Ser^ variants into *D. melanogaster*. *miniwhite*: visible *miniwhite^+^* eye marker to identify transgenic flies. FRT: flippase recognition target. direction of triangle signals the directionality of the FRT. tRNA^Ser^: tRNA^Ser^_UGA_-1-1 variant with ~300 bp of native up- and downstream sequence. MCS: multiple cloning sequence with *Not*I site removed. *attB*: *attB* component of *attB/attP* attachment sites used in ΦC31-integrase recombination. *AmpR*: ampicillin resistance gene.


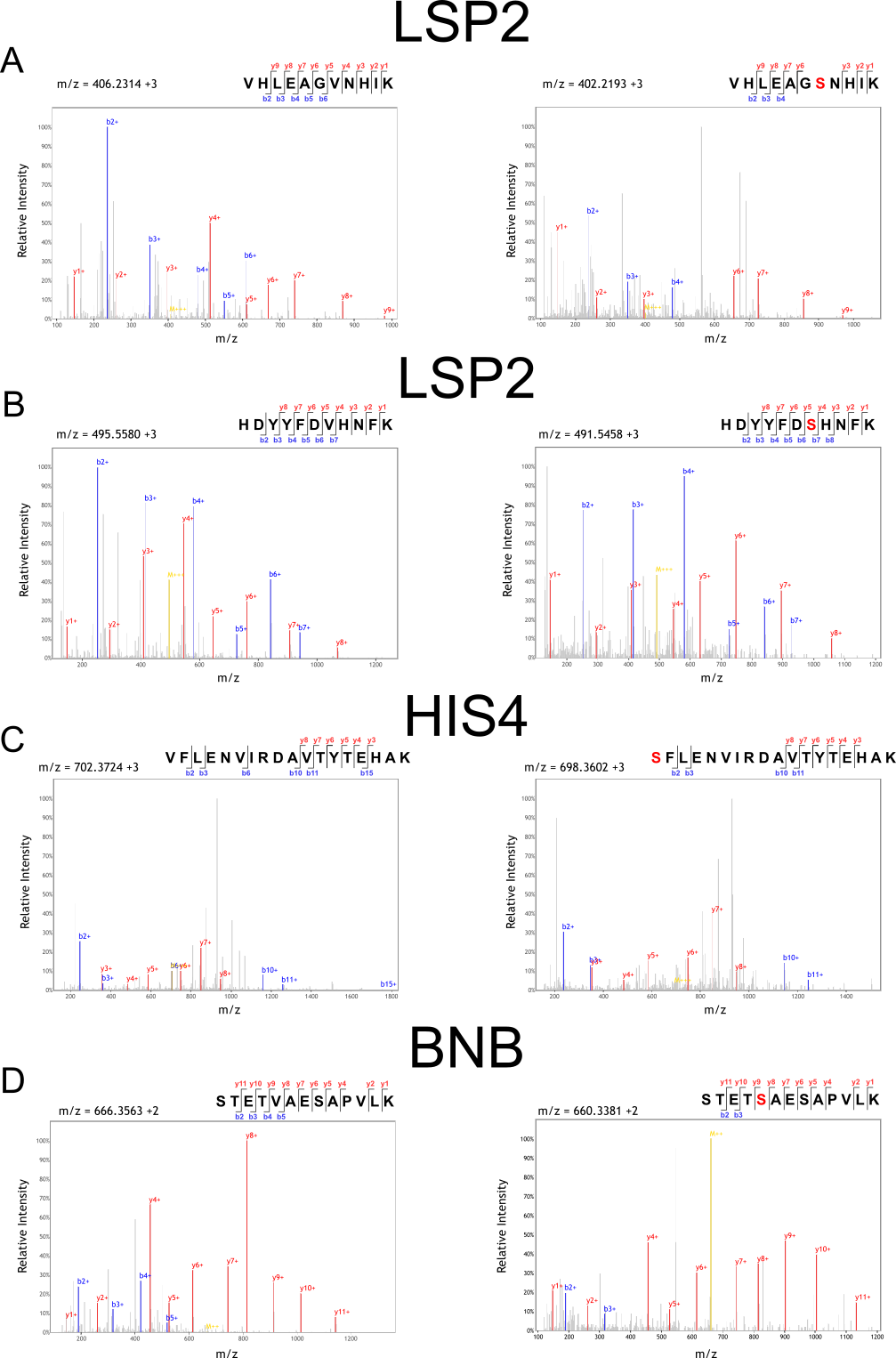


**Figure S2.** *Representative spectra of peptides containing a V🡪S substitution in ≥ 4 of 5 tRNA^Ser^_AAC_ adult replicates*. Spectra of a wild-type (left) or V🡪S mistranslated peptide (right) corresponding to **A)** and **B)** LSP2, **C)** HIS4, **D)** BNB. Mistranslated peptides were identified in at least four of five mistranslating adult tRNA^Ser^_AAC_ (V🡪S) replicates but were absent in all control tRNA^Ser^_AAC_-FLP (control) replicates.


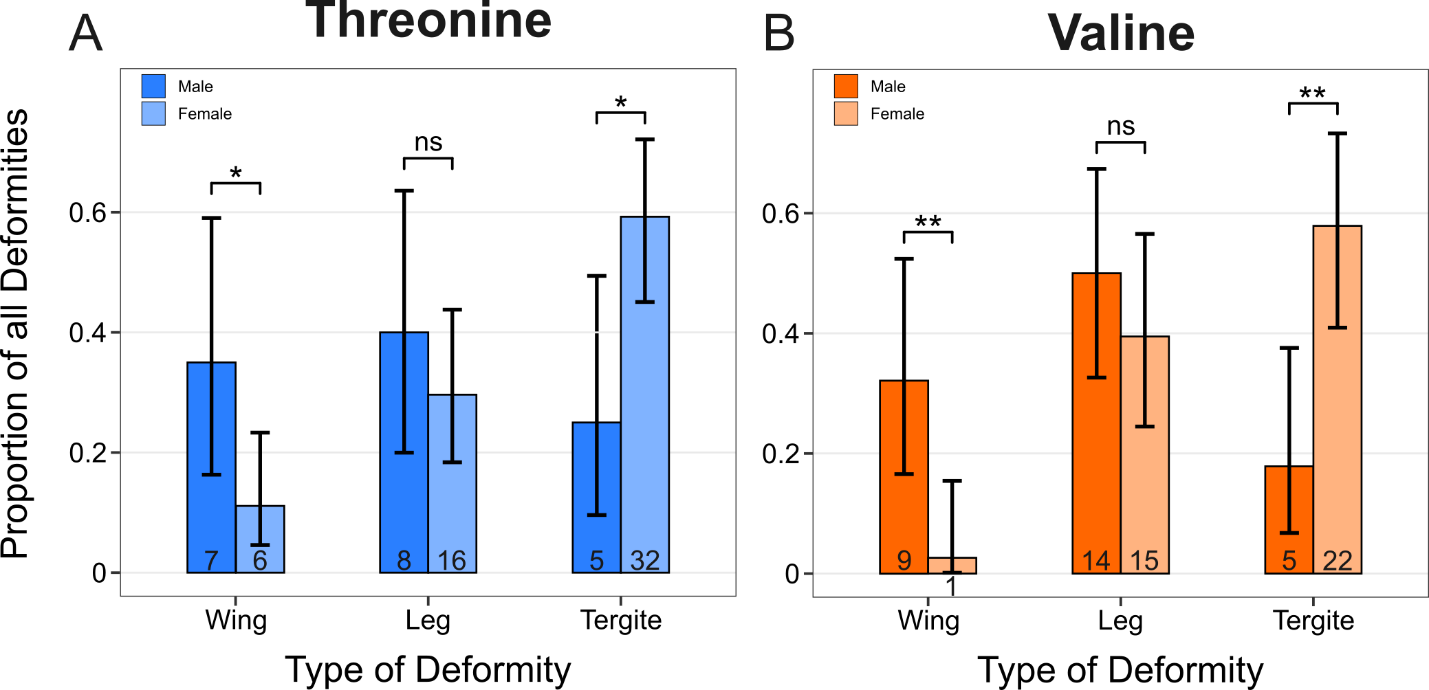


**Figure S3.** *Proportion of each type of scored deformity in male and female T🡪S or V🡪S flies*. The proportion of wing, leg, or tergite deformities out of all deformities observed in male or female flies containing **A)** tRNA^Ser^_AGU_ (T🡪S) or **B)** tRNA^Ser^_AAC_ (V🡪S). Frequency of each deformity were compared to the opposite sex using a chi-square test with post-hoc correction as outlined in Shan and Gerstenberger (2017). “ns” *P* ≥ 0.05, “*” *P* < 0.05, “**” *P* < 0.01.


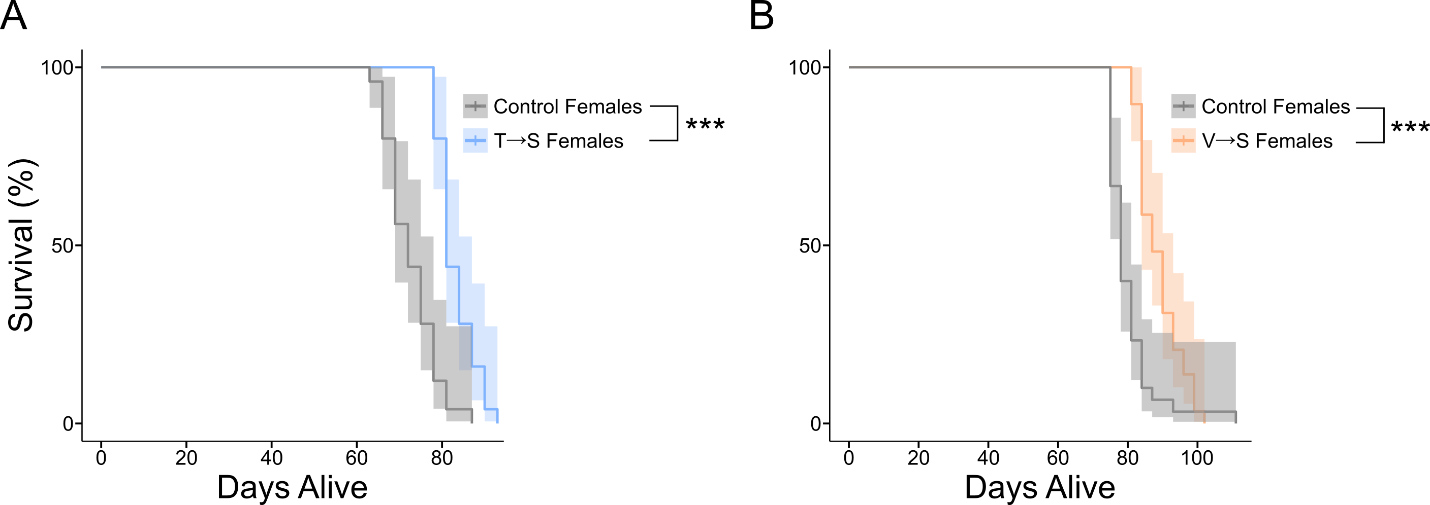


**Figure S4.** *Comparing survival of the top 25% longest-lived females from control and mistranslating lines.* **A)** Kaplan-Meier survival curves constructed from the 25 longest-lived tRNA^Ser^_AGU_ (T🡪S) or tRNA^Ser^_AGU_-FLP (control) females. Shaded region representing the 95% confidence interval of survival probability. **B)** Kaplan-Meier survival curves constructed from the 30 longest-lived tRNA^Ser^_AAC_ (V🡪S) or tRNA^Ser^_AAC_-FLP (control) females. Survival curves were statistically compared using log-rank tests corrected using Holm-Bonferroni’s method. “***” P < 0.001.


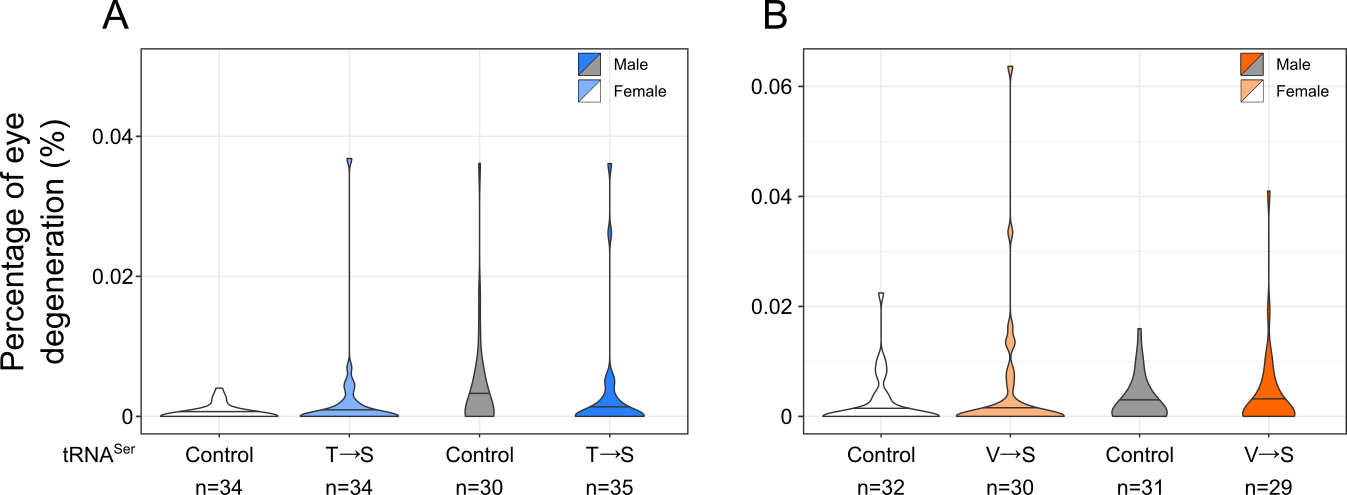


Figure S5. *Amount of eye degeneration in tRNA^Ser^_AGU_ (T🡪S) and tRNA^Ser^_AAC_ (V🡪S) compared to control flies*. A) Violin plot showing the distribution of the amount of eye degeneration in a 15-ommatidia radius of the left fly eye belonging to male or female tRNA^Ser^_AGU_ (T🡪S) or tRNA^Ser^_AGU_-FLP (control). Eye degeneration was calculated as the number of pixels corresponding to degenerated areas divided by the total scored area of the eye. Numbers below the genotype label represent the sample size. B) Same as A) but for tRNA^Ser^_AAC_ (V🡪S) and tRNA^Ser^_AAC_-FLP (control) flies. There were no significant differences between groups according to Wilcoxon rank-sum tests corrected using Holm-Bonferroni’s method.

**
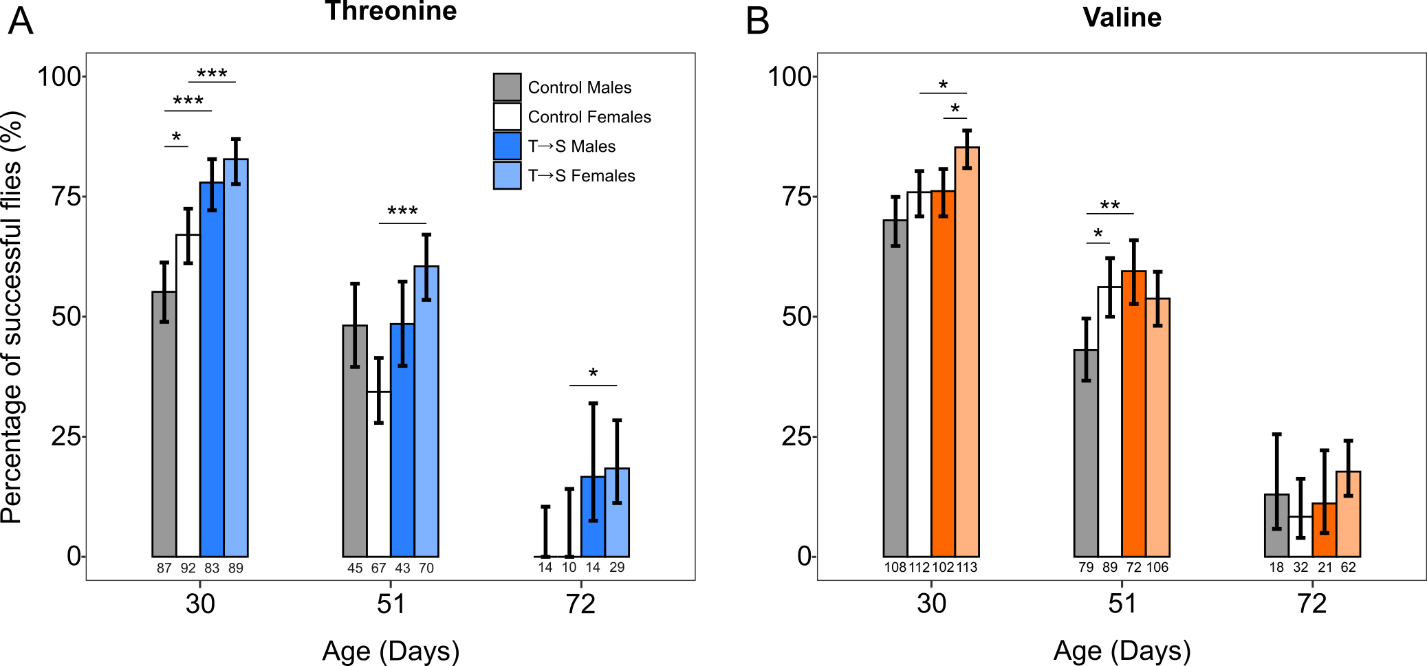
Figure S6**. *Climbing performance of tRNA^Ser^_AGU_ (T🡪S) and* ***B)*** *tRNA^Ser^_AAC_ (V🡪S), including deformed flies*. Each bar represents the percentage of flies from the specified genotype that successfully reached a 5 cm goal line in 10 seconds. All flies were tested three times. The numbers below the bars represent the number of flies of that genotype and age that were tested. **A)** Climbing performance of tRNA^Ser^_AGU_ (T🡪S) or control (tRNA^Ser^_AGU_-FLP) male and female flies at 30, 51, and 72 days of age. **B)** Climbing performance of tRNA^Ser^_AAC_ (V🡪S) or control (tRNA^Ser^_AAC_-FLP) male and female flies at 30, 51, and 72 days of age. Performance was compared between groups using Fisher’s exact test and *P*-values were corrected for multiple comparisons using Holm-Bonferroni’s method. Error bars represent the 95% confidence interval of the proportion. Only significant comparisons are shown. “*” *P* < 0.05; “**” *P* < 0.01; “***” *P* < 0.001.

**Literature Cited**

Chan, P. P., and T. M. Lowe, 2016 GtRNAdb 2.0: An expanded database of transfer RNA genes identified in complete and draft genomes. Nucleic Acids Res 44: D184–D189.

Eden, E., R. Navon, I. Steinfeld, D. Lipson, and Z. Yakhini, 2009 GOrilla: a tool for discovery and visualization of enriched GO terms in ranked gene lists. BMC Bioinformatics 10: 48.

Shan, G., and S. Gerstenberger, 2017 Fisher’s exact approach for post hoc analysis of a chi-squared test. PLoS One 12: e0188709.
